# New and Reemerging Infectious Diseases

**DOI:** 10.3201/eid0908.030324

**Published:** 2003-08

**Authors:** Roberto Docampo

**Affiliations:** University of Illinois at Urbana-Champaign, Urbana, Illinois, USA

The Sixth Annual Conference on New and Reemerging Infectious Diseases was hosted April 24–25, 2003, by the Center for Zoonoses Research and the College of Veterinary Medicine, University of Illinois at Urbana-Champaign (UIUC). The conference featured seven speakers and 27 poster presentations.

## Smallpox

Bertram L. Jacobs (Arizona State University, Tempe, AZ) opened the conference with a presentation on smallpox, one of the most devastating diseases known to humankind. Smallpox was eradicated from the wild in the 1970s, although the potential use of Variola virus as a bioterrorism agent makes it still of great concern. Dr. Jacobs described Vaccinia viruses deficient in E3L, a regulator of the cellular antiviral response and noted their potential for the production of improved vaccines. He also showed that double-stranded (ds)RNA- and ZDNA binding proteins had a role in poxvirus pathogenesis. In the poster section, Joanna Shisler (University of Illinois at Urbana-Champaign [UIUC], Urbana) reported that the modified virus, Ankara, activates nuclear factor κB through the mitogen-activated protein kinase, extracellular signal–regulated kinase (MEK)/extracellular signal–regulated kinase (ERK) pathway, possibly facilitating the host immune response. This virus was used to vaccinate 100,000 people, with no reported complications, at the end of the global smallpox vaccination campaign led by the World Health Organization in the 1970s.

## West Nile Virus and Geographic Information Systems

Since it was first detected in New York City in 1999, West Nile virus (WNV) has spread from coast to coast and has been found in 43 states from Maine to California. Stephen C. Guptill (U.S. Geological Survey, Reston, VA) reported that the U.S. Geological Survey is working with the Centers for Disease Control and Prevention (CDC) to learn the current geographic extent of WNV. This will allow us to understand how it moves between birds, mosquitoes, and humans and to better predict future outbreaks. A collaborative 3-year research project is being conducted on lands administered by the U.S. Fish and Wildlife Service, the National Park Service, and other federal lands, and on state, local, and private lands along the Atlantic and Mississippi flyways. This study tests sampled migratory and local wild birds to detect WNV and identify possible avian carriers. Over 10,000 birds of more than 150 species have been captured, sampled, and released at 20 federal sites and 3 other sites in 12 states during the spring and fall bird migration seasons of 2001 and 2002. A parallel study, conducted with CDC, is examining the distribution and number of mosquito species in relation to land cover, weather conditions, and avian deaths. Systematic mosquito surveillance (weekly collections at seven sites) is being conducted year-round in St. Tammany Parish in Louisiana, complementing avian collections at the Bogue Chitto and Big Branch National Wildlife Refuges in the parish. Finally, WNV surveillance data from CDC is being studied to determine the spatial and temporal relationships between disease outbreaks in birds and animals and human illness. Information from these analyses will guide the creation of predictive models of disease risk. These surveillance systems provide the basic information on the “geography” of the virus. Combining these data with information about avian migratory patterns, landscape characteristics, and weather conditions, over space and time, will provide the foundation for developing spatial analytical and forecasting models to assess the risk for human illness. In related work, presented at the poster session, Marylin Ruiz (UIUC, Urbana) reported the efforts of the College of Veterinary Medicine Geographic Information System and Spatial Analysis Laboratory, in collaboration with the Illinois Department of Public Health, and the Illinois Department of Agriculture in the mapping and analysis of the WNV outbreak in Illinois. (Illinois was the state hit the hardest by the epidemic in 2002.) Geographic information systems in conjunction with fine resolution satellite data and spatial statistics are also useful to investigate the distribution of other diseases, for example, schistosomiasis (Julie A. Clennon, UIUC, Urbana).

## Animal Models of Infectious Diseases

Streptococcal pathogens continue to evade concerted efforts to decipher clear-cut virulence mechanisms, although numerous genes have been implicated in pathogenesis. Melody N. Neely (Wayne State University, Detroit, MI) reported the development of a unique animal model, the zebrafish (*Danio rerio*), to characterize specific virulence mechanisms used within various tissues in vivo. Her group is using this model host to study infection by two streptococcal species that represent two forms of streptococcal disease: a natural pathogen of fish and humans, *Streptococcus initiae,* and a human-specific pathogen, *S. pyogenes*. *S. initiae* primarily causes a fatal systemic disease in the zebrafish after intramuscular injection, with pathologic changes similar to those seen in human infections caused by *S. agalactiae* and *S. pneumoniae*. The fatal infection by *S. pyogenes* causes a locally spreading necrotic disease confined to the muscle with pathologic features similar to those observed in a human infection of necrotizing fasciitis. By studying pathogens that are virulent for both fish and humans and that mediate disease states in the zebrafish identical to those found in human streptococcal infections, common virulence strategies shared by a number of gram-positive pathogens can be identified. Using several genetic strategies with the two streptococcal strains, Dr. Neely’s group is currently conducting specific screens in the zebrafish to 1) identify and characterize cell membrane proteins that interact with the host in vivo to cause specific disease states; 2) identify genes required for growth in vivo, as well as progressive stages of infection; 3) identify genes that are only expressed while in the host along with tissue specificity of the encoded proteins; and 4) analyze responses of the host that affect progression of disease.

## Foodborne Diseases

*Shigella boydii* is a food pathogen that was implicated in a 1999 foodborne outbreak involving contaminated bean salad that contained fresh parsley and cilantro. Hans Blaschek and collaborators (UIUC, Urbana) reported the high tolerance of this bacterium to acidic pH, and the presence and formation of biofilms in cilantro and parsley samples treated with produce wash and water. Cells in biofilms are known to be more resistant to antibiotics and disinfectants, and biofilm formation may explain the decreased efficacy of produce wash on parsley and cilantro samples.

## Intracellular Parasites

The trypanosomatid protozoan *Leishmania* synthesizes a variety of glycosylphosphatidylinositol (GPI)**–**anchored molecules on its surface. Sorting out their individual functions has been difficult and in some cases controversial as they share structural domains and generally have been tested outside the context of key components such as the major glycolipid lipohosphoglycan (LPG) and phosphoglycans (PGs); ether lipids, which constitute approximately 20% of the membrane lipids and most GPI anchors; and others. Stephen M. Beverley and his collaborators (Washington University, St. Louis, MO) have studied the role of GPI-anchored proteins role in the context of parasite knockouts and “add-back” controls, probing their role in diverse aspects of parasitism such as entry, inhibition of phagolysosomal fusion and host-signal transduction, and pathogenesis. All of these molecules play critical roles in virulence, although sometimes in unanticipated ways. PGs in particular are required for parasite persistence but not acute pathology, therefore defining a new class of parasite genes important to transmission. Ted Hackstadt (National Institute of Allergy and Infectious Diseases, National Institutes of Health, Hamilton, MT) reported that, unlike most intracellular parasites, which block maturation of endosomes to lysosomes at discrete stages and then replicate within those vacuoles, chlamydiae appear to dissociate themselves from the endocytic pathway shortly after internalization by actively modifying the vacuole to become fusogenic with sphingomyelin-containing exocytic vesicles. Interaction with a secretory pathway appears to provide a pathogenic mechanism that allows chlamydiae to establish themselves in a site not destined to fuse with lysosomes. Fusion with Golgi-derived vesicles provides a likely source of cellular lipids for the growth of the inclusion membrane as it expands to accommodate the multiplying parasites.

Kasturi Haldar (Northwestern University, Chicago, IL) presented studies on the malaria parasite, focusing on protein trafficking, gene expression, and drug development. Her group has studied vacuolar trafficking of host raft proteins and parasite virulence determinants (by tagging genes with green fluorescent protein and expressing them by transfection) for their consequences on malarial entry into the red blood cell, virulence secretion systems, and apicoplast biogenesis. The apicoplast is a newly identified residual plastid acquired by secondary endosymbiosis that has attracted attention for its evolutionary novelty and its potential as a drug target. Temporal regulation of plasmodial genes may be important for protein- targeting in cells. In this context, Dr. Haldar’s group is examining the role of unique promoter elements and chromatin in regulating expression of secretory determinants such as the histidine-rich proteins and adherence antigens. Whole genome scanning approaches (microarrays and other functional approaches) are being used in combination with informatics to develop novel lipid-linked targets for drug development. Studies on *Salmonella* are currently examining effectors of the SPI-2 system (*Salmonella* Pathogenicity Island–2 system for their effects on sterol recruitment, metabolism, and bacterial virulence in the mouse model. The requirement for nonsterol precursors in protecting infected cells from death by apoptosis, necrosis, or both, is also being investigated. Finally, microarrays are used to identify subsets of *S. typhimurium* virulence determinants required for lipid-linked intracellular bacterial replication.

*Toxoplasma gondii* is a major cause of birth defects and infections in immunocompromised persons. Like all members of the phylum Apicomplexa, *T. gondii* is an obligate intracellular organism. Micronemes and rhoptries are specialized secretory organelles of the Apicomplexa, whose contents are thought to be essential for successful invasion of host cells. Kami Kim (Albert Einstein College of Medicine, Bronx, NY) reported identifying two subtilisin-like serine proteinases from *T. gondii*, TgSUB1 and TgSUB2, which are necessary for successful invasion. Serine proteinase inhibitors have been reported to block host cell invasion by both *T. gondii* and the related apicomplexan parasite, *P. falciparum*. Disruption of *TgSUB2* was unsuccessful, which implies that *TgSUB2* is an essential gene. Both TgSUB1 and TgSUB2 undergo autocatalytic processing as they traffic through the secretory pathway. TgSUB1 is a microneme protein, whereas TgSUB2 localizes to rhoptries and associates with rhoptry protein ROP1, a potential substrate. Mutational analysis suggests that TgSUB2 is a rhoptry protein maturase. Processing of secretory organelle contents appears to be ubiquitous among the Apicomplexa. Since subtilases are found in genomes of all the Apicomplexa sequenced to date, they may represent a novel chemotherapeutic target. Transcriptional regulatory pathways in apicomplexan parasites are understudied and may contain novel drug targets. William Sullivan and his collaborators (Indiana University School of Medicine, Indianapolis, IN) identified and mapped a gene in *T. gondii* that encodes a homologue of chromatin remodeling factors that uses ATP to promote a more favorable environment for transcription. They also described a novel histone acetyltransferase, which contains a unique 820-amino acid *N*-terminal extension of unknown function.

## Parasite Organelles

Acidocalcisomes are novel calcium-containing acidic organelles present in unicellular eukaryotes. Several posters from researchers in the groups of Silvia Moreno and Roberto Docampo (UIUC, Urbana) highlighted recent work on these organelles. Andrea Montalvetti described a functional aquaporin (water channel) found in the organelles of *T. cruzi*, the etiologic agent of Chagas disease, and Peter Rohloff showed that this protein is translocated to the contractile vacuole upon hypo-osmotic stress. Joanna Cox and Shuhong Luo demonstrated that a Ca2^+^-ATPase is localized to acidocalcisomes and the plasma membrane of *Trypanosoma brucei*, the etiologic agent of African sleeping sickness, and is essential for cell growth, as demonstrated by RNA interference experiments. Manfredo Seufferheld presented evidence of the presence acidocalcisomes in the bacterium *Rodospirillum rubrum*. Felix Ruiz showed that acidocalcisomes of *Plasmodium falciparum*, one of the etiologic agents of malaria, do not differ significantly from the organelles in other apicomplexan parasites, whereas the platelet-dense granules possess several characteristics common to acidocalcisomes. *T. brucei* possesses a P-type proton ATPase with homology to fungal and plant H^+^-ATPases but absent in mammalian cells; this proton pump constitutes a formidable target for chemotherapy (Shuhong Luo, UIUC, Urbana)

## Chemotherapeutic Targets

A number of poster presentations identified novel chemotherapeutic targets for infectious diseases. Steve Grimme (UIUC, Urbana), who received an award for the best poster presentation, demonstrated that a GPI mannosyltransferase (CaSMP3) was essential for the human pathogenic fungus *Candida albicans* and therefore was validated as a potential target for chemotherapy. M. Laura Salto (UIUC, Urbana) showed that inositolphosphoceramide, a lipid used in anchoring several proteins to the plasma membrane of *T. cruzi* and absent in the host, is remodeled during the differentiation of different stages of the parasite. Novel lipid modified phosphoinositide phospholipases C were reported in *T. cruzi* (Michael Okura, UIUC, Urbana) and *T. brucei* (Jianmin Fang, UIUC, Urbana). The enzymes are apparently involved in differentiation of the parasites and are different from their host counterparts. Recently, bisphosphonates have been proposed as novel antiparasitic drugs, and Yan Ling (UIUC, Urbana) reported the cloning and characterization of *Toxoplasma gondii* farnesyl pyrophosphate synthase, the target of these compounds. Tryptophan metabolism in mosquitoes and the separation and identification of mosquito chrorion proteins through two-dimensional electrophoresis and mass spectrometry will provide useful targets against these vectors of several infectious diseases, as reported by Jianyong Li and his collaborators (UIUC, Urbana).

